# HIV-1 Infection and the PPAR*γ*-Dependent Control of Adipose Tissue Physiology

**DOI:** 10.1155/2009/607902

**Published:** 2008-12-01

**Authors:** Marta Giralt, Pere Domingo, Francesc Villarroya

**Affiliations:** ^1^Department of Biochemistry and Molecular Biology and Institute of Biomedicine, University of Barcelona, 08028 Barcelona, Spain; ^2^CIBER Fisiopatología de la Obesidad y Nutrición, Instituto de Salud Carlos III, 08028 Barcelona, Spain; ^3^Department of Internal Medicine, Hospital de la Santa Creu i Sant Pau, 08025 Barcelona, Spain

## Abstract

PPAR*γ* is a ligand-dependent master transcription factor controlling adipocyte differentiation as well as multiple biological processes taking place in other cells present in adipose tissue depots such as macrophages. Recent research indicates that HIV-1 infection-related events may alter adipose tissue biology through several mechanisms involving PPAR*γ*, ranging from direct effects of HIV-1-encoded proteins on adipocytes to the promotion of a proinflammatory environment that interferes with PPAR*γ* actions. This effect of HIV-1 on adipose tissue cells can occur even in the absence of direct infection of adipocytes, as soluble HIV-1-encoded proteins such as Vpr may enter cells and inhibit PPAR*γ* action. Moreover, repression of PPAR*γ* actions may relieve inhibitory pathways of HIV-1 gene transcription, thus enhancing HIV-1 effects in infected cells. HIV-1 infection-mediated interference of PPAR*γ*-dependent pathways in adipocytes and other cells inside adipose depots such as macrophages is likely to create an altered local environment that, after antiretroviral treatment, leads to lipodystrophy in HIV-1-infected and HAART-treated patients.

## 1. Introduction

A complex set of metabolic alterations, preferentially involving adipose tissue, has emerged in recent years in a substantial number of HIV-1-infected patients under highly-active antiretroviral treatment (HAART). This is the so-called HIV-1/HAART-associated lipodystrophy syndrome. Disturbances in adipose tissue of these patients range from lipoatrophy of subcutaneous adipose tissue to visceral adipose accumulation or lipomatosis [1]. Moreover, during recent years, basic research on adipose tissue biology has succeeded in identifying major molecular players in the differentiation and function of adipose tissue. Among them, the peroxisome proliferator-activated receptor *γ* (PPAR*γ*)subtype of PPARs has emerged as a master transcriptional regulator of adipose cells [2]. Although we are still far from a full understanding of the molecular basis of HIV-1 lipodystrophy, research has been actively undertaken in this area and has examined the role of alterations in PPAR*γ*-dependent pathways in eliciting the syndrome [3, 4]. Regardless of the potential effects of antiretroviral drugs on PPAR*γ*, several recent findings suggest that HIV-1 infection-related events may cause disturbances in the PPAR*γ*-dependent pathways of control of adipose tissue physiopathology, and they are summarized in the present review.

## 2. Adipose Tissue, HIV-1 Infection, and Lipodystrophy

The concepts concerning the causal basis of HIV-1 associated lipodystrophy have evolved substantially in the last few years. The initial identification of HIV-1 lipodystrophy was coincident with the introduction of drugs from the protease inhibitor family for use in antiretroviral treatment, and these drugs were at first considered to be causative of the syndrome [5]. After it was recognized that patients without protease inhibitor treatment could also develop lipodystrophy, the syndrome was associated with HAART overall. Although several drugs of HAART regimes are known at present to be more prone to induce the appearance of lipodystrophy or of some of their particular features (abdominal fat hypertrophy versus peripheral lipoatrophy) than others [6], no single class of drugs can account for elicitation of the overall syndrome. On the other hand, there have been suggestions that antiretroviral treatment causes lipodystrophy only when acting upon HIV-1-infected patients, and that events related to HIV-1 infection are intrinsically associated with the development of the syndrome. Evidently, there are no data on long-term antiretroviral treatment of non-HIV-1-infected patients that could establish the specific role of HAART independent of the HIV-1 infection, and a single two-week study of nucleotide-analog reverse transcriptase inhibitor treatment of non-infected volunteers indicated the appearance of only a few features of the lipodystrophy syndrome [7].

Some data have indicated that mild alterations of adipose tissue biology are already present in nontreated HIV-1-infected patients [8]. The studies of body composition in the era before HAART reported a disproportionate loss of body fat mass in men with advanced HIV-1 disease or AIDS [9]. This was attributed to the progression towards AIDS-related wasting and associated diseases. However, some data suggested that weight loss and depletion of body fat may precede the progression to AIDS, even in adults with normal CD4+ lymphocyte counts [10]. Further studies confirmed an indirect effect of HIV-1 viremia leading to effects similar to AIDS wasting. Studies by Visnegarwala et al. of HIV-1-infected men with CD4+ lymphocyte counts >200 cells/mm^3^ suggested that malnutrition due to decreased caloric intake and increased energy demands associated with an active opportunistic infection was not contributing factors in explaining reduced body weight [11]. It was concluded that reduction in fat mass is related to HIV-1 infection itself, independent of the additive effects of opportunistic illnesses. Similar conclusions were obtained from the analysis of dyslipemia. Before the introduction of HAART, the early appearance of hypertriglyceridemia and enhanced lipolysis was observed in HIV-1-infected patients before the onset of overt illness [12, 13]. Recent studies have confirmed that HIV-1 infection-induced changes in lipolysis are unrelated to the further effects of HAART leading to full-blown lipodystrophy [14]. Finally, several rodent models relevant to HIV-1 infection events have shown metabolic disturbances in the absence of any exposure to antiretroviral drugs. For instance, transgenic mice expressing the HIV-1 accessory viral protein R (Vpr) in liver and adipose tissue exhibit altered systemic fat metabolism [15]. Disturbances of PPAR*γ*, as a master regulator of adipose tissue differentiation and function, may play a major role in the alterations of adipose mass and lipid metabolism elicited by HIV-1 infection, and this issue is summarized in the present review.

## 3. PPAR*γ* and Its Pivotal Role in Adipose Tissue Function

PPAR*γ* is highly expressed in adipose tissue, where it plays a key role in adipose tissue development and function. There are two major splice variants of PPAR*γ*, *γ*1 and *γ*2, which differ in their N-terminal region (PPAR*γ*2 contains an additional 30 amino acids) and in their tissue-specific expression; PPAR*γ*2 is found almost exclusively in white and brown adipose tissues whereas PPAR*γ*1 is also relatively abundant in macrophages and endothelial cells [16–18]. The activity of both isoforms is regulated by posttranscriptional modifications and by ligand-dependent transactivation and recruitment of coactivators. For instance, phosphorylation inhibits the transcriptional activity of PPAR*γ* [19] and promotes sumoylation, which further reduces its transcriptional activity [20]. PPAR*γ* forms heterodimers with retinoid *X* receptors (RXRs) to bind to specific DNA sequences in its target genes. In the absence of ligands, corepressors such as nuclear receptor corepressor (N-CoR) or silencing mediator of retinoid and thyroid (SMRT) receptors bind to these heterodimers and recruit histone deacetylases to repress transcription (reviewed in [21]). Binding of ligands to PPAR*γ* triggers conformational changes that allow the recruitment of transcriptional coactivators, including members of the steroid receptor coactivator (SRC) family [22] and PPAR*γ*-coactivator 1*α* (PGC-1*α*) [23] that ultimately recruit histone acetyltransferase coactivators such as p300/CBP or PCAF [21].

However, the natural ligands for PPAR*γ* remain unknown. Recent studies have provided functional evidence for an unidentified natural ligand that is produced transiently during adipogenesis [24]. There is also evidence that small lipophilic compounds, such as polyunsaturated fatty acids and fatty acid derivatives (eiocosanoids) bind and activate this receptor [25], thus supporting the concept that PPAR*γ* is a nutrient sensor that finely regulates metabolic homoeostasis in response to different nutritional states.

Regarding synthetic ligands, it is clear that members of the thiazolidinedione (TZD) family of antidiabetic drugs are high-affinity agonists for PPAR*γ* [26]. TZDs have been reported to enhance insulin sensitivity in animals and humans [27]. Furthermore, cellular, genetic, and pharmacological studies have provided strong evidence both that TZDs function via PPAR*γ*, and that adipose tissue is the main site where the insulin-sensitizing effects of PPAR*γ* are produced (reviewed in [28]).

It was reported that TZDs induce adipocyte differentiation even before they were known to be ligands of PPAR*γ* [29]. By now, the key role of PPAR*γ* as a master regulator of adipogenesis has been clearly established, and gain-of-function experiments have demonstrated that PPAR*γ* is sufficient to induce adipocyte differentiation in the presence of an appropriate ligand [30]. However, loss-of-function experiments to prove that PPAR*γ* is required for this process have been more difficult, since PPAR*γ* homozygous inactivation results in embryonic death due to placental alteration, in a developmental stage before there is any adipose tissue development [31]. Later, however, studies utilizing chimeric mice [32] and adipose-specific PPAR*γ* knockout mice [33] confirmed the essential role of PPAR*γ* in adipose tissue differentiation.

PPAR*γ* also plays an important role in regulation of lipid metabolism in mature adipocytes. Activation of PPAR*γ* increases both fatty acid uptake and its storage into adipocytes by promoting the transcription of genes such as those encoding lipoprotein lipase, fatty acid binding protein-4 (aP2/FABP4), phosphoenolpyruvate carboxykinase (PEPCK) [34–37], and also glucose transporter GLUT-4, in order to increase fatty acid synthesis [38]. These effects of PPAR*γ* may underlie its insulin-sensitizing effects. Thus, together with the proadipogenic role of PPAR*γ* (glucose homeostasis requires adequate amounts of adipose tissue), the improvement of lipid storage in this tissue will prevent ectopic lipid accumulation in nonadipose tissues such as liver, skeletal muscle, and *β*-cells. Furthermore, PPAR*γ* has been reported to induce transcription of the PGC-1*α* gene in adipose tissue [39]. The coactivator PGC-1*α* promotes mitochondrial biogenesis, thus leading to an increase in fatty acid oxidation in adipose tissue, which may protect against adipocyte hypertrophy [40]. Finally, adipose tissue has endocrine functions, and PPAR*γ* regulates expression of genes encoding adipokines such as adiponectin, leptin, resistin, or cytokines such as TNF*α*. Activation of PPAR*γ* promotes the expression of a pro-insulin-sensitizing adipokine profile (i.e., induction of adiponectin and reduction of TNF*α* gene expression) thus involving the cross-talk between adipose tissue and other insulin-sensitive organs (liver, skeletal muscle) in the insulin-sensitizing effects of PPAR*γ* [41].

Evidence from human mutations in PPAR*γ* has further underlined the importance of PPAR*γ* in the development of adipose tissue, in the maintenance of glucose and lipid homeostasis and more generally in the control of energy balance (reviewed in [42]). Patients harboring mutations in the ligand-binding domain of PPAR*γ* have a stereotyped phenotype characterized by partial lipodystrophy, severe insulin resistance, dyslipidemia, hepatic steatosis, and hypertension, thus identifying PPAR*γ* as playing a molecular role in the pathogenesis of the metabolic syndrome [43, 44].

PPAR*γ* is also expressed in macrophages and endothelial cells, that is, cells that are present in adipose tissue [17, 18, 45]. In endothelial cells, activation of PPAR*γ* has antiproliferative, antiangiogenic, and anti-inflammatory effects [45]. PPAR*γ* is induced during macrophage differentiation, and its activation increases the expression of macrophage-specific markers, such as CD14 and CD11b [17, 46]. However, loss-of-function approaches have demonstrated that PPAR*γ* is not essential for monocyte/macrophage differentiation either in vivo or in vitro [47, 48] but selective deletion of PPAR*γ* in macrophages results in increased insulin resistance [49]. Recently, macrophage-mediated inflammation in adipose tissue has been proposed to play a central role in the pathogenesis of insulin resistance [50]. Two types of macrophages, proinflammatory M1 and anti-inflammatory M2, are present in adipose tissue and their relative abundance may change dynamically through recruitment of polarized monocytes from the blood (macrophage infiltration) or through the effects of local cytokines on macrophages in adipose tissue. Activation of PPAR*γ* by TZDs has now been reported to increase the proportion of anti-inflammatory M2 macrophages in adipose tissue [51]. Furthermore, TZDs also act through PPAR*γ* to inhibit the expression of inflammatory mediators in macrophages, and as reported above, to negatively regulate expression of cytokines such as IL-6, TNF-*α*, and monocyte chemoattractant protein—1 (MCP-1/CCL-2) in adipocytes [52]. In summary, activation of PPAR*γ* improves adipose tissue function by having a beneficial effect on the adipocyte—macrophage relationship, which may result in prevention of insulin resistance.

## 4. HIV-1 Infection and PPAR*γ*

Recent studies revealed that expression of marker genes of adipogenesis, such as those encoding GLUT-4, adiponectin, or lipoprotein lipase is already altered in subcutaneous adipose tissue from HIV-1-infected patients in the absence of treatment [53]. These genes are known targets of PPAR*γ*, and the expression of PPAR*γ* itself is also reduced in HIV-1-infected patients without treatment, relative to healthy controls [53]. In fact, in this same sense, it has been observed that PPAR*γ* expression is lower in HIV-1-infected and HAART-treated patients with lipodystrophy relative to healthy controls [54], but similar when compared to levels in antiretroviral-naive patients [55]. These findings point to the possibility that HIV-1 infection and associated events interfere with the action of PPAR*γ* as a master transcriptional controller of adipogenesis and, in a broader sense, of adipose tissue biology, thus contributing to the appearance of lipodystrophy. Recent experimental evidence is supportive of this possibility (see below). However, a relevant role of HAART in worsening potential HIV-1-mediated alterations in PPAR*γ* expression cannot be excluded. In this sense, it has been recently reported that a 6-month interruption of antiretroviral treatment results in a significant amelioration of PPAR*γ* levels in patients formerly under HAART containing protease inhibitors [56]. On the other hand, one of the features of HIV-1-associated lipodystrophy is insulin resistance. It cannot be excluded that PPAR*γ* impairment in HIV-1-infected patients may contribute to reduced insulin sensitivity, as PPAR*γ* is a known target of drugs with insulin-sensitizing properties (see above).

It must be taken into account that the action of HIV-1 infection in adipose tissue and PPAR*γ* activity should not be considered only in relation to adipocytes. As stated above, adipose tissue depots contain, in addition to adipocytes, other cells such as preadipocytes, macrophages, or endothelial cells. Recently, even lymphocytes have been reported to be present inside adipose depots [57, 58]. As these other cell types (e.g., macrophages or endothelial cells) also express PPAR*γ*, they could themselves be sensitive to HIV-1-mediated disturbances in PPAR*γ* expression and activity. Moreover, cells inside adipose tissue can release regulatory factors (such as adipokines or cytokines) or metabolites (free fatty acids) capable of influencing surrounding cells, for instance preadipocytes and adipocytes, and the overall pathways of gene regulation dependent on PPAR*γ*.

## 5. How May HIV-1 Infection Affect PPAR*γ* Pathways in Adipose Tissue?

The possibility that HIV-1 infection could influence PPAR*γ* activity leads to the first consideration; whether the cells present in adipose tissue depots can be infected by HIV-1. The capacity of HIV-1 to infect adipocytes is controversial. Whereas some initial reports indicated that adipocytes could be infected [59, 60], later data appeared to exclude this possibility [61]. However, more recently, it was reported that substantial infection of adipocytes could take place when TNF*α* was present in the medium [62]. High levels of TNF*α* are found in adipose tissue from HIV-1-infected patients even before treatment and are part of a proinflammatory environment already present in adipose tissue as a consequence of long-term HIV-1 infection [53, 63]. It should be noted that studies on the capacity of HIV-1 to infect adipose cells have focused on mature adipocytes and less is known concerning preadipocytes. In adipose depots, resident preadipocytes are found and they can differentiate into mature adipocytes. Lipoatrophic situations combine a loss of adipose cells (primarily via apoptosis) and an incapacity of preadipocytes to differentiate and replenish the depot; thus any direct or indirect effect of HIV-1 infection that interferes with PPAR*γ* could lead to this impaired differentiation. Recent identification of PPAR*γ* gene mutations causative of lipodystrophies of genetic origin supports the notion that abnormal PPAR*γ* function can lead to lipodystrophy [42, 64].

Evidently, if a cell of the adipocyte lineage is infected by HIV-1, it will be exposed to gene products of HIV-1 and to their potential effects on PPAR*γ* actions. A reported example of this is Nef, a 27 kDa HIV-1-encoded protein that localizes in the cytoplasm as well as nucleus of infected cells [65]. It has been shown that nuclear Nef results in a reduction in the expression of PPAR*γ* and of PPAR*γ* gene targets in human T cells and macrophages as well as interfering with fat accumulation in cell lines [66]. The effects of Nef were specific in impairing PPAR*γ*-dependent, but not PPAR*α*-dependent, transcriptional activity.

However, the most relevant evidence of interference of PPAR*γ* pathways by HIV-1-encoded products concerns the HIV-1 accessory protein Vpr. Kopp and collaborators have recently shown that Vpr suppresses the differentiation of adipocytes in cell culture by interfering with PPAR*γ*-dependent transactivation of target genes [67]. Vpr acts as a corepressor of PPAR*γ* by interacting with the ligand-binding domain of the receptor in an agonist-dependent manner. Remarkably, this effect could be observed when Vpr was added to the adipose cell culture media thus indicating that, as already shown in other cell types [68], exogenous Vpr can enter the cell and interfere with metabolic pathways. These findings are highly relevant to an understanding of the etiopathogenesis of lipodystrophy in HIV-1-infected patients. Thus, it is expected that adipose cells can be affected by Vpr either due to direct infection by HIV-1, or indirectly, because Vpr is known to be present as a soluble protein in the circulation of HIV-1 infected patients [69]. Moreover, adipocytes and preadipocytes may be exposed to local concentrations of Vpr higher than those in the overall circulation, given the proximity of cells such as macrophages (or even resident lymphocytes) that can be infected and release Vpr.

Finally, recent research has revealed several features of the biology of PPAR*γ*, in relation to adipogenesis, in which HIV-1 infection may be hypothesized to interfere. Thus, several players in cell cycle control, such as cyclin D3 and CDK4, have been reported to promote adipogenesis through interaction with PPAR*γ* [70, 71], whereas E2F4 represses PPAR*γ* during adipogenesis [72]. HIV-1 infection may interfere with the cell cycle machinery, and specifically the HIV-1-encoded proteins Vpr and Tat have been recognized as being capable of interacting with CDK4, cyclin D3, and E2F4 [73–75]. However, the capacity of these interactions to influence PPAR*γ*-dependent pathways in adipocytes or in other cells present in adipose depots, and their consequences for metabolism, remains to be explored. Similarly, a number of reports have indicated that HIV-1-encoded Tat or Vpr can interact with known coactivators of PPAR*γ* required for its transcriptional activity, such as p300/CBP or PCAF [76, 77]. It cannot be excluded that HIV-1 infection-mediated events affect PPAR*γ* activity in adipose tissue through these interactions, although experimental evidence for this is lacking at present.

## 6. The Reciprocal Issue: the Effect of PPAR*γ* on HIV-1 Biology, and the Implications of This for Adipose Tissue Pathophysiology

What we have described up to now provides evidence that HIV-1 infection may alter PPAR*γ* activities. However, several reports also indicate the occurrence of reciprocal events, that is, the action of PPARs and particularly PPAR*γ* on HIV-1 biology, especially on the replication and transcription of the HIV-1 genome. The capacity of nuclear receptors to interact with the long-terminal repeat of HIV-1 was recognized several years ago [78]. It was observed that heterodimers of RXR and PPAR*α* were capable of binding a region between −356 to −320 in the long terminal repeat. Accordingly, PPAR*α* agonists such as clofibric acid were shown to activate HIV-1 transcription [79]. This effect, which may be relevant in tissues such as liver which express high levels of PPAR*α*, is not expected to be involved in alterations of white adipocytes and preadipocytes which are almost devoid of PPAR*α* [80].

In contrast, it was reported recently that the PPAR*γ* agonist ciglitazone inhibits HIV-1 replication in a dose-dependent manner in acutely-infected human monocyte-derived macrophages and in latently-infected and viral entry-independent U1 cells, suggesting an effect at the level of HIV-1 gene expression [81]. Cotransfection of PPAR*γ* wild-type vectors and treatment with PPAR*γ* agonists inhibited HIV-1 promoter activity in U937 cells, and activation of PPAR*γ* also decreased HIV-1 mRNA stability following actinomycin D treatment. Thus, natural and synthetic PPAR*γ* agonists may play a role in controlling HIV-1 infection in macrophages [81, 82]. Similar observations were obtained by Skolnik et al. who observed that activation of PPAR*γ*, and also of PPAR*α*, by specific agonists also decreased HIV-1 replication in peripheral blood mononuclear cells acutely infected with HIV-1, in a chronically-infected monoblastoid cell line and in alveolar macrophages from HIV-1-infected subjects and uninfected controls [83]. The precise mechanisms of action of PPAR*γ* on HIV-1 are not fully known and, in addition to the potential direct interaction with specific regions of the long-terminal repeat mentioned above, indirect effects via nuclear factor *κ*B have also been proposed on the basis of the effects of PPAR*γ* and its ligand rosiglitazone impairing nuclear factor *κ*B-mediated enhancement of HIV-1 replication in macrophages [84].

All these findings indicate the occurrence of a potential cross-talk between PPAR*γ* and HIV-1 that could reinforce the activity of HIV-1 proteins in cells harboring PPAR*γ*. Thus, a reduction in PPAR*γ* levels and/or activity as a consequence of HIV-1 infection may lead to depression of such a pathway of potential inhibition of HIV-1 transcription and could create an environment prone to enhancement of HIV-1 gene expression, establishing a “vicious cycle” further augmenting adipose pathogenesis.

## 7. Conclusions

**Figure 1:**
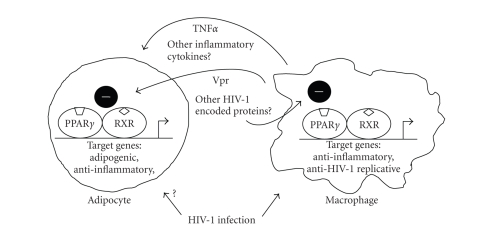
Schematic representation of the potential effects of HIV-1 infection on PPAR*γ*-mediated pathways in adipocytes and macrophages. HIV-1 infection of macrophages may lead to the synthesis of HIV-1 encoded proteins, that is, Vpr, with negative effects on the expression of PPAR*γ* target genes. This may lead to reduced expression of anti-inflammatory genes as well as promotion of HIV-1 replication. Release of HIV-1-encoded proteins as well as enhanced production of inflammatory cytokines, that is, TNF*α* and other, by macrophages as a consequence of HIV-1 infection may lead to impaired PPAR*γ* action in adipocytes and preadipocytes, thus impairing adipogenesis and fat accretion. Direct effects of HIV-1 infection in line with what happens in macrophages cannot be excluded. Similar events to those depicted for macrophages could be considered to occur in endothelial cells or even lymphocytes present in adipose tissue depots as a consequence of HIV-1 infection.

In summary, research to date indicates that HIV-1 infection-related events may alter adipose tissue and contribute to development of the full-blown lipodystrophy syndrome after antiretroviral treatment. The role of HIV-1 infection of cells in adipose tissue, of soluble proteins released by infected cells as well as of the indirect effects elicited by the mild proinflammatory environment associated with viral infection, is issues expected to be the subject of intense research in the near future. For all these aspects, PPAR*γ* appears as a main candidate for the mediation of pathogenic events. Moreover, a full understanding will be required of the relationships among the complex set of cell types that, in addition to adipocytes, are present in adipose tissue depots. Macrophages, endothelial cells, preadipocytes, and perhaps even lymphocytes are present in adipose depots and establish a complex regulatory cross-talk that is altered by HIV-1 infection, and which may ultimately lead to disturbances in adipocytes and in the whole adipose mass (see Figure 1). All of these cell types express PPAR*γ*, and the pivotal role of this receptor in adipogenesis, insulin sensitivity, lipid metabolism, and inflammatory pathways point to this receptor as a key target of future research on adipose tissue disturbances in the HIV-1/HAART-associated lipodystrophy syndrome.
